# Cochrane Rapid Reviews Methods Group to play a leading role in guiding the production of informed high-quality, timely research evidence syntheses

**DOI:** 10.1186/s13643-016-0360-z

**Published:** 2016-10-28

**Authors:** Chantelle Garritty, Adrienne Stevens, Gerald Gartlehner, Valerie King, Chris Kamel, on behalf of the Cochrane Rapid Reviews Methods Group

**Affiliations:** 1 Ottawa Methods Centre, Ottawa Hospital Research Institute, 501 Smyth Road, Ottawa, Ontario K1H 8L6 Canada; 2 Translational Research in Biomedicine (TRIBE) Graduate Program, University of Split School of Medicine, Šoltanska 2, 21000 Split, Croatia; 3 Cochrane Austria, Danube University, Krems, Dr. Karl Dorrek Strasse 30, 3500 Krems, Austria; 4 RTI International, 3040 East Cornwallis Rd, Research Triangle Park, NC 27709 USA; 5 The Center for Evidence-based Policy, Oregon Health & Science University, 3030 SW Moody Avenue, Portland, OR 97201 USA; 6 Canadian Agency for Drugs and Technologies in Health (CADTH), 600-865 Carling Ave., Ottawa, Ontario K1S 5S8 Canada

**Keywords:** Rapid reviews, Knowledge synthesis, Evidence synthesis, Healthcare decisions

## Abstract

**Background:**

Policymakers and healthcare stakeholders are increasingly seeking evidence to inform the policymaking process, and often use existing or commissioned systematic reviews to inform decisions. However, the methodologies that make systematic reviews authoritative take time, typically 1 to 2 years to complete. Outside the traditional SR timeline, “rapid reviews” have emerged as an efficient tool to get evidence to decision-makers more quickly. However, the use of rapid reviews does present challenges. To date, there has been limited published empirical information about this approach to compiling evidence. Thus, it remains a poorly understood and ill-defined set of diverse methodologies with various labels. In recent years, the need to further explore rapid review methods, characteristics, and their use has been recognized by a growing network of healthcare researchers, policymakers, and organizations, several with ties to Cochrane, which is recognized as representing an international gold standard for high-quality, systematic reviews.

**Purpose:**

In this commentary, we introduce the newly established Cochrane Rapid Reviews Methods Group developed to play a leading role in guiding the production of rapid reviews given they are increasingly employed as a research synthesis tool to support timely evidence-informed decision-making. We discuss how the group was formed and outline the group’s structure and remit. We also discuss the need to establish a more robust evidence base for rapid reviews in the published literature, and the importance of promoting registration of rapid review protocols in an effort to promote efficiency and transparency in research.

**Conclusion:**

As with standard systematic reviews, the core principles of evidence-based synthesis should apply to rapid reviews in order to minimize bias to the extent possible. The Cochrane Rapid Reviews Methods Group will serve to establish a network of rapid review stakeholders and provide a forum for discussion and training. By facilitating exchange, the group will strive to conduct research to advance the methods of rapid reviews.

## Background

Cochrane is an international non-profit organization that is the world’s largest producer of high-quality systematic reviews (SRs) of effectiveness with over 37,000 contributors from more than 130 countries. This work is managed by 53 review groups that span a range of health topics. A SR typically takes at least 12 months to conduct. However, the SR process can range anywhere from 6 months to 2 years [1–3]. Ensuring the methodological rigor of the SR process is labor-intense. As a consequence, SRs often do not meet the time-sensitive needs of some users, notably healthcare policymakers. A more timely process is required to respond to pressured decision-making. The international knowledge synthesis community has responded by developing rapid reviews (RRs), described as members of the knowledge syntheses family [4]. RRs are thought to be more systematic than narrative reviews, use abbreviated SR methods, and are completed within shorter timeframes ranging from a few weeks to usually no more than 6 months. RRs can take different forms along a spectrum of products with various labels such as rapid evidence synthesis, rapid evidence assessment, or rapid evidence summary to name a few. Common to these products is that they require methodological shortcuts that may increase the uncertainty of findings.

Policymakers are using RRs in their day-to-day decision-making, and RRs have been shown to be influential on the development of health policy [2, 5–10]. Further, there are strong signals of increased use among researchers and other healthcare stakeholders [1, 2, 10–13]. The existence of at least 29 international organizations that conduct RRs has been formally documented [13], and anecdotally we know of more. Further, due to the increased interest of public authorities and clinicians, both the World Health Organization (WHO) and the Guidelines-International-Network (G-I-N) have undertaken methods for developing guidelines in an accelerated timeframe using RR methods to inform recommendations [14–16].

While the concept RR is not novel, it remains a poorly understood and as yet ill-defined set of diverse methodologies supported by a paucity of published, available scientific literature. For example, there is no universally accepted definition of RR. Of the definitions that do exist [2, 17–21], not all address variances in types of RRs produced across different contexts, and most appear to be driven by a particular mandate or scope of the entities producing and commissioning them. Although we consider the main difference between SRs and RRs to be methodological shortcuts, some would argue that a SR delivered within accelerated timelines should be considered a RR. The challenge is that with no clear or formal definition of RR, this issue has yet to be addressed. It may be the case that when doing a SR rapidly, some decisions may be made under pressure including use of methodological shortcuts in order to meet a deadline. However, it is unclear how often this might occur and how much the potential impact is on the final products. Another factor to consider is whether a SR was conducted quickly because the scope was narrow and/or the existing literature base to review was minimal compared with a SR that was sizeable but with additional resources allocated to meet a short timeline. Thus, further methodological research is needed to inform this discussion.

A minimum set of consensus-based criteria as to what constitutes a RR will be instrumental in guiding future research discussions and is needed to improve transparency and understanding by end-users. Some investigations have examined the characteristics of RRs to better understand what methods are being employed and to what extent they resemble similar conduct stages of a SR [1, 10, 11, 22–25]. However, little is known about when to undertake RRs, whether they provide similar results to SRs, and the impact of methodological shortcuts (relative to the SR process) on the validity of results and subsequent conclusions. Further, RRs are challenging to identify in that once produced or commissioned by organizations, they are often not published in peer-reviewed journals. Of those that are published, unless the terms “rapid (systematic) review” and “rapid evidence assessment or synthesis” are in the title or abstract, it can be difficult to classify such studies given the various synonyms for RR. None of the mainstream biomedical bibliographic databases such as MEDLINE®/PubMed® currently employ a specific indexing term for the RR publication type. It is important to note that the methodological choices when doing a RR in order to meet the specific needs of a commissioning body within a short timeframe might subsequently restrict the scope, usefulness, and reusability of the review. As with any report, the perceived quality of a RR may impede publication in a peer-reviewed journal.

In spite of their challenges, the speed with which RRs are gaining prominence and are being incorporated into urgent decision-making underscores the need to further explore their methods, characteristics, and use. While RR producers must answer the time-sensitive needs of the healthcare decision-makers they serve, they must simultaneously ensure that the scientific imperative of methodological rigor is satisfied. In order to adequately address this inherent tension, a need for methodological research and development of standards has been identified [26].

For these reasons, and with interest from within the SR community, we have established a Cochrane methods group to better inform “rapid review” methodology that will serve the Cochrane membership and beyond and will provide an opportunity for Cochrane to position itself as a leader in this emerging field of synthesis, akin to the influence it has had on SRs.

### The role and function of the Cochrane Rapid Reviews Methods Group

The notion of developing a RR methods group within Cochrane germinated from the 2013 Cochrane Colloquium in Quebec City, Canada, when a core of the current convenors planned and facilitated an information-sharing session open to interested participants. The colloquium also included an oral session that featured a series of focused presentations on RRs. These two colloquium events, which drew much attention, indicated there was interest in RR methods and their applications, particularly as they relate to health policy and decision-making. Several themes were raised including reasons that would constitute why RRs are undertaken rather than traditional SRs. Further, various organizations in attendance at the open discussion contributed information on their respective RR program methodology, including how they define “rapid review”, types of reports produced, and specific steps involved in topic selection, protocol development, report production, and report submission and dissemination. From these exploratory events, an informal network of researchers and healthcare organizations involved with and, or interested in, RRs materialized. Over the next 2 years, additional exploratory discussions were held, namely at the Canadian Cochrane Symposium (2014) and Evidence Live in the UK (2015) to further assess interest and support for a methods group. These efforts culminated in the registration of the Cochrane Rapid Reviews Methods Group in October 2015, now the newest of the 17 global Cochrane Method Groups for those with interest and expertise in the science of SRs.

At the present time, the role of Cochrane Rapid Reviews Methods Group is to actively involve a large community of relevant stakeholders to engage in methods innovations and development in rapid evidence synthesis. Specifically, our group will serve as a discussion forum; be involved in RR methods research, development, and evaluation; lead RR methods guidance and handbooks; and produce standards for the conduct and reporting of RRs. As the group strives to develop a valued network for people to share and develop ideas in relation to RRs, it will be important that it maintain a database of RR publications and of persons with interest in RRs, and to establish linkages with other organizations and potential commissioners or end-users of RRs. This Cochrane group will also endeavor to deliver training through Cochrane events and elsewhere. Whether a RR producer, commissioner, funder, or SR methodologist, our hope is to encourage you to join and participate in this important work.

Presently, the Cochrane Rapid Reviews Methods Group is comprised of five co-convenors from Canada, the USA, and Austria. Although unfunded, the group is sustained through institutional in-kind support provided by the Ottawa Hospital Research Institute (OHRI), Cochrane Austria, the Center for Evidence-based Policy at Oregon Health & Science University (OHSU), and the Canadian Agency for Drugs and Technologies in Health (CADTH). Virtual co-administration is provided by the Ottawa Methods Centre based at OHRI, and Cochrane Austria. As we launch the start of the group, we are encouraged by our current membership, which consists of over 250 individuals from 30 countries. For further information about joining our membership, or to learn more about the group, visit the Cochrane Rapid Reviews Methods Group website at http://methods.cochrane.org/rapidreviews/welcome.

### Establishing a more robust evidence base for rapid reviews

We recognize that, to date, a limited number of RRs have been published in the peer-reviewed literature, with most reports found by searching the gray literature space. Moreover, even fewer studies specific to RR methods studies have been published. However, since the start of 2015, there has been an increase in the number of relevant references aiming to advance the knowledge of RRs including aspects of conduct and the perception of stakeholders. The willingness of *Systematic Reviews* to publish RR-related articles [2, 4, 13, 22, 25–33] has provided an important platform for disseminating developments to facilitate timely production of RRs. We are encouraged to see more published contributions to the field of RRs and look forward to seeing additional key studies published in *Systematic Reviews* and elsewhere, and how they may contribute to our unfolding work as a Cochrane methods group.

### Promoting efficiencies

At a time of rising healthcare costs, restricted research funding, and the need to reduce waste in research, RRs are on the radar as a useful tool helping to inform evidence-based healthcare decision-making. In order to reduce the risk of unplanned duplication of RRs, the Cochrane Rapid Reviews Methods Group encourages all producers and commissioners of RRs to consider registering their RR protocol with PROSPERO, the international prospective register of SRs, including Cochrane reviews [34]. To date, only a small fraction of PROSPERO’s more than 10,000 records are registered as “rapid”, according to listed titles and abstracts. One reason could be that the notion of registration is still unknown to many researchers. Another might be that RR is not yet an official PROSPERO category of “review type”, although currently under consideration. Nonetheless, RRs are eligible if they meet PROSPERO’s eligibility criteria (http://www.crd.york.ac.uk/PROSPERO/). Registration would allow those commissioning or planning RRs to identify whether any RRs on a given topic are underway, to avoid unintended duplication of effort and to increase transparency of methods. Therefore, the Cochrane Rapid Reviews Methods Group will actively promote registration as part of its remit. Cochrane’s involvement with RRs is currently being explored but is yet to be determined. As a result, although RRs are not formally incorporated into Cochrane, the Cochrane Rapid Reviews Methods Group aims, at this stage, to connect with the broader research synthesis community in order to make RR methods development more visible internationally.

## Conclusions

Although important differences exist between developing a standard SR and RR, it will be important that the core principles of evidence-based synthesis apply to RRs to minimize bias to the extent possible given accelerated timeframes, applying a transparent process, and use of explicit methods. Therefore, establishment of a methods group such as this one will be important in guiding methods research in order to mitigate the challenges that plague RRs, in spite of the purpose they serve.

## Abbreviations

RRs: Rapid reviews
SRs: Systematic reviews
